# Optical spatial modulation design

**DOI:** 10.1098/rsta.2019.0195

**Published:** 2020-03-02

**Authors:** T. Cogalan, H. Haas, E. Panayirci

**Affiliations:** 1 University of Edinburgh, Li-Fi R&D Centre, Edinburgh EH9 3JL, UK; 2 Kadir Has University, Department of Electrical and Electronics Engineering, Istanbul 34083, Turkey

**Keywords:** light-fidelity, multiple-input multiple-output, spatial modulation, intensity modulation and direct detection, pulse amplitude modulation, multi-user multiple-input multiple-output

## Abstract

Visible light communication (VLC) systems are inherently signal-to-noise ratio (SNR) limited due to link budget constraints. One favourable method to overcome this limitation is to focus on the pre-log factors of the channel capacity. Multiple-input multiple-output (MIMO) techniques are therefore a promising avenue of research. However, inter-channel interference in MIMO limits the achievable capacity. Spatial modulation (SM) avoids this limitation. Furthermore, the performance of MIMO systems in VLC is limited by the similarities among spatial channels. This limitation becomes particularly severe in intensity modulation/direct detection (IM/DD) systems because of the lack of phase information. The motivation of this paper is to propose a system that results in a multi-channel transmission system that enables reliable multi-user optical MIMO SM transmission without the need for a precoder, power allocation algorithm or additional optics at the receiver. A general bit error performance model for the SM system is developed for an arbitrary number of light-emitting diodes (LEDs) in conjunction with pulse amplitude modulation. Based on this model, an LED array structure is designed to result in spatially separated multiple channels by manipulating the transmitter geometry.

This article is part of the theme issue ‘Optical wireless communication’.

## 1. Introduction

Visible light communication (VLC) is becoming a promising technology for indoor environments by using light-emitting diodes (LEDs) for illumination as well as data transmission simultaneously. The concept of visible light communications (VLC) is extended to light-fidelity (Li-Fi) technology that is conceived as a light-based bi-directional multi-user wireless network which supports user mobility. Signal transmission in a Li-Fi system is performed by using intensity modulation (IM), which is a technique to convey information on the instantaneous optical power at the transmitter side. At the receiver side, direct detection (DD) is used to convert the received optical intensity to an information signal-dependent photo-current. As a consequence, the transmit signal should be real-valued and non-negative. Therefore, well-studied radio frequency (RF)-based techniques may not be applied straightforwardly to IM/DD Li-Fi systems.

A system with multiple elements at the transmitter and receiver is known as a multiple-input multiple-output (MIMO) whereas multiple transmit elements with a single receive element is called a multiple-input single-output (MISO) system. In RF systems, MIMO and MISO have been extensively studied to enhance the system capacity by simultaneous transmission from all transmit elements. It has been shown that increasing the number of transmit and/or receive elements substantially improves the achievable data rates by increasing the *spatial multiplexing* gain [1,2].

Achieving the *spatial multiplexing* gain is possible with an appropriately constructed transmission matrix. This is known as *precoding* and the statistical properties of the RF channel due to fading are used to spatially separate the multiple channels in MIMO and MISO systems [3,4]. In other words, *precoding* is employed at the transmitter to mitigate correlation or inter-channel interference between the transmit elements. In IM/DD systems, channel gains rely on the geometry between the transmitter and receiver, hence, they are subject to a strong deterministic component. The similarities^1^ among spatial channels severely limit the performance of MISO/MIMO systems due to the lack of phase information. Having spatially separated multi-element channels for IM/DD systems relies on the appropriate design of the transmitter and/or receiver. Thus, in this study, MISO/MIMO systems are introduced under two categories, namely multiplexing MIMO and imaging MIMO. In the first category, the design is carried out at the transmitter and both RF and IM/DD systems are discussed. For IM/DD systems, a non-imaging photo diode (PD) is used as the receiver. In the second category, only IM/DD-based MIMO systems that use some optical processing at the receiver in addition to the transmitter design are discussed.

### (a) Multiplexing multiple-input multiple-output

In general, obtaining the inverse or eigenvectors of the channel matrix is required to design a precoder. Hence, *precoding* becomes computationally complex especially when the number of transmit and receive elements is high. Moreover, simultaneous transmission from all transmit elements increases power consumption. Alternatively, the inter-channel interference problem in MISO/MIMO systems can also be mitigated by the use of spatial modulation (SM). In SM, only a single transmit element is activated during a symbol transmission period where the index of the activated element, known as the spatial symbol, conveys extra information [4,5].^2^ Therefore, SM provides a good trade-off between the achievable rate and power consumption/energy efficiency. However, as the index of the activated element conveys information, correlation/similarities among the transmit elements plays a key role in the bit error ratio (BER) performance of SM systems.

The BER of the SM systems is studied in detail for generalized RF fading channels in [6]. It is shown that the spatial channel correlation affects the distance between the spatial constellation points, which in turn degrades the BER performance of the system. In [7], the minimum distance between the SM symbols is maximized by a transmit precoding algorithm. The precoding algorithm in [7] jointly finds the optimum Euclidean distance between all the received signals instead of maximizing the minimum distance. It is shown in [7] that optimizing the Euclidean distance between the received symbols outperforms the BER of the precoder, which maximizes the minimum distance.

Indoor MISO or MIMO IM/DD systems have been studied in [8–15] and in [16–19] in conjunction with SM. It is shown that the optical multi-transmit element channel have close similarities and, specifically, employing SM relies on the appropriate design of the transmitter and receiver [16,18,19]. The similarity between optical channels is reduced by power allocation algorithms which are proposed as part of the transmitter design in [10–13,19]. In [12,13], in addition to the power allocation algorithm, the orientation of LEDs in an LED array is used to mitigate channel similarities among the multi transmit elements. Whereas in [9,14,20], the orientation of non-imaging PDs at the receiver, which is known as an angle diversity receiver, is used to reduce the channel similarities. It is shown in [12–14,20] that changing the geometry of the transmitter and/or receiver can result in a spatially separated multi-element channel.

### (b) Imaging multiple-input multiple-output

Two types of imaging MIMO system exist in the literature: optics based and camera based. In optics-based imaging MIMO, an optical component such as lens is used to image each transmit LED onto a detector array that consists of pixels [8,21–23]. In camera-based imaging MIMO, a camera image sensor is used to convert incident light on sensor pixels to voltage [24,25]. In [8], in addition to the non-imaging MIMO system, the performance of an imaging MIMO system is investigated and the experimental results are shown. More investigations have been carried out in [21] to improve the performance of the non-imaging MIMO system proposed in [8]. In [22], an imaging receiver is designed along with a custom transmitter to investigate the feasibility of achieving spatially separated channels. An imaging receiver structure that uses a hemispherical lens is proposed in [23] to mitigate the similarities among a multi-element channel. Although the proposed optics-based imaging MIMO systems reduced the similarities between optical channels, there are some limitations by means of implementation such as an increase in receiver size, additional optics and a smaller field-of-view (FOV) which reduces the probability of having a line of sight (LoS) link. For the camera-based imaging MIMO systems, again, spatially separated channels can be achieved but there are performance limitations due to the perspective distortions, blurs, frame rate and shutter speed of the camera.

In this study, the geometry dependency of the channel gain in the IM/DD systems is used to simplify the transmitter complexity by manipulating the transmitter geometry. Instead of designing a precoder, power control algorithm and/or a receiver with some optics, a novel transmit LED array structure along with multiple simple LEDs and non-imaging PDs at the receiver is proposed. The proposed system results in a spatially separated multi transmit element channel. The optical SM studies presented in [16,17,20] consider multiple LED luminaires located as a grid on the ceiling of a room. Although a grid-based LED luminaire placement is reasonable for office environments, most of the indoor environments such as living rooms and bedrooms are not suitable for this deployment. Moreover, all of the deployed LEDs cannot be used for SM in every location in a grid-based LED luminaire placement. In some locations in the indoor environment, the channel gain from the LEDs that are located away from each other is almost zero. Employing SM in such a deployment and using all of the deployed LEDs results in a rank deficient channel matrix. Therefore, in a grid-based LED deployment, a centralized unit is needed to employ SM in order to (i) choose LEDs that have a high channel gain; and (ii) provide data to the chosen LEDs. However, in this study, a single LED luminaire, consisting of multiple LEDs is considered to employ SM and to provide a required illumination level throughout the entire indoor environment. Firstly, the relationship between the error probability and the channel similarity of an optical 2-pulse amplitude modulation (PAM) 2 × 1 MISO-SM system given in [19] is extended to a 2-PAM 4 × 1 MISO-SM system. Then, based on the findings when using an increased number of transmit LEDs, a generalized relation between channel similarity and symbol error probability is evaluated for an arbitrary number of transmit elements and PAM order *M* when the channel gain of the transmit elements follows some specific properties. According to the generalized relationship, an LED array structure design is proposed to separate multiple channels by manipulating the transmitter geometry without the need to employ either a power allocation algorithm or a precoder. For the sake of comparison, the system model considered in [16] is adapted in this paper, and the performance of a unipolar *M*-ary PAM MIMO-SM system is investigated when the proposed transmitter structure is employed along with simple non-imaging PDs at the receiver. In addition, when there are more than one user present in the system, the error probability becomes dependent not only on the channel similarity of the transmit elements of a single user but also the channel similarity among transmit elements of all users. Therefore, the multi-user MIMO-optical SM system becomes interference-and-similarity-limited, instead of solely noise-and-similarity-limited. Hence, a transmit element selection procedure is defined for both single and multiple user SM transmission and the proposed LED array structure is examined.

The paper is organized as follows. The system model is described in detail in §2. The relation between the channel gains and error performance for a SM system with an arbitrary number of LEDs and *M*-ary PAM is given in §3. In §4, the channel model and the design of the proposed transmit LED array structure are described. How to select LEDs for single and multi-user transmissions is given in §5. The simulation parameters and results are presented in §6. Finally, §7 concludes the paper.

*Notation*: Throughout the paper, vectors and matrices are written in bold lower-case and upper-case letters, respectively. The transpose and Frobenius norm of a vector is expressed by (.)^T^ and $||.|{|}_{\text{F}}$, respectively. Real normal distribution is given by $N(\mu ,\sigma^{2}),$ where *μ* represents mean and *σ*^2^ is variance. $R^{+}$ denotes the ring of positive real numbers. The argument of the minimum and maximum are represented by arg⁡min{.} and arg⁡max{.}, respectively.

## 2. System model

**Figure 1.**
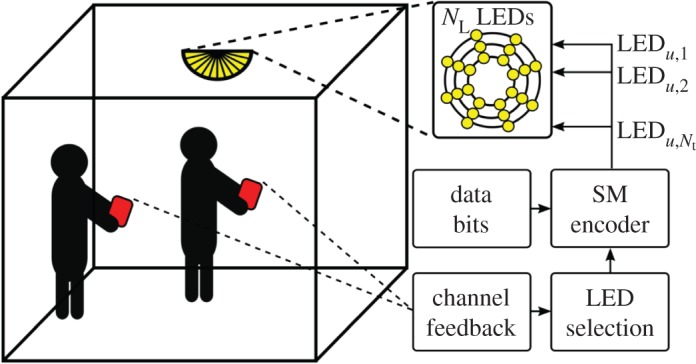
Multi-user optical SM system model and block diagram. The LED array consists of *N*_L_ LEDs and *N*_t_ of them are chosen for each user for SM transmission. LED_*u*,*t*_ represents the *t*^th^ LED that is selected for user *u*. (Online version in colour.)

A multi-user optical MIMO system depicted in figure 1 is considered. At the transmitter, an LED array with multiple LEDs, consisting of *N*_L_ LEDs, is used. *N*_u_ multiple users, with *N*_p_ multiple PDs, are assumed at different locations inside the room. It is assumed that the LED array is located in the middle of a room and the channel state information (CSI) of the users is fed-back to the transmitter. Based on the CSI of each user, *N*_t_ of *N*_L_ LEDs are chosen for each user *u* ∈ {1, …, *N*_u_} for the transmission of data using an optical SM encoder which employs unipolar *M*-PAM. The chosen set of LEDs for a user *u* is represented by $L^{u}≜\{{\text{LED}}_{u,t}\}$ where LED_*u*,*t*_ is the LED index *i* ∈ {1, …, *N*_L_} in the set of all LEDs $N_{\text{L}}≜\{1,\ldots ,N_{\text{L}}\}$; and *t* ∈ {1, …, *N*_t_}.

Accordingly, the received signal **y** of a user *u* can be written in vector form as follows:

2.1 $$y_{u}={\hat{H}}_{u}s_{u}+n_{u},$$

where ${\hat{H}}_{u}\in R_{N_{\text{p}}\times N_{\text{t}}}^{+}$ is the optical channel gain matrix of user *u*, which is a sub-matrix of $H_{u}\in R_{N_{\text{p}}\times N_{\text{L}}}^{+}$; $s_{u}\in R_{N_{\text{t}}\times 1}^{+}$ is the transmitted signal vector for user *u*; and $n_{u}\in R_{N_{\text{p}}\times 1}$ is the additive white Gaussian noise (AWGN) vector where each element of **n**_*u*_ is independently distributed with $N(0,\sigma^{2})$. In this paper, without loss of generality, the optical-to-electrical (O/E) and electrical-to-optical (E/O) conversion coefficients are assumed to be one.

When SM is employed in a system, data is encoded both as spatial and PAM constellation symbols. Since only one of the LEDs is activated during transmission, the overall spectral efficiency of the system is log _2_(*N*_t_) + log _2_(*M*) bits/s/Hz. As noted in [16], a signal constellation with zero intensity cannot be used in optical SM, due to the fact that there would not be an active transmit element and hence no spatial information exists based on SM principles. In this paper, the intensity levels of *M*-PAM signals for optical SM are given as

2.2 $$I_{m}^{M}=A\left(2m-1\right),\;\text{for}\;m=1,2,\ldots ,M,$$

where *A* = *I*/*M*; and *I* is the average emitted optical power. The definition in (2.2) is different from the one used in [16]. In [16], the intensity levels are chosen such tat the distance between the levels is smaller than the levels given in (2.2). Therefore, it can be expected that the bit error probability of the system is higher in [16] for the same intensity range and exactly the same link conditions. The intensity level of the transmit LED is decided according to the constellation symbol, and the transmit LED is activated based on the spatial symbol.

**Figure 2.**
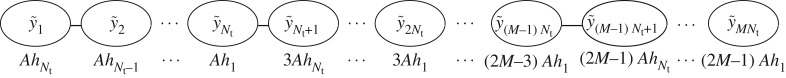
Received symbols constellation in the absence of noise. *A* is *I*/*M*.

Interested readers are referred to [16, fig. 2] for more details on the described *M*-PAM-optical SM model.

## 3. Minimum error performance

As the spatial symbol decides which LED is activated to transmit the *M*-PAM symbol, the similarity among *N*_t_ transmit LEDs directly effects the error performance of an optical SM system. In SM systems, the spatial and *M*-PAM constellation symbols are jointly estimated by the maximum-likelihood (ML) detector at the receiver as follows [16]:

3.1 $${\hat{s}}_{u}=\arg\max_{s_{u}}p_{y_{u}}(y_{u}|s_{u},{\hat{H}}_{u})=\arg\min_{s_{u}}||y_{u}-{\hat{H}}_{u}s_{u}|{|}_{\text{F}}^{2},$$

where $p_{y_{u}}$ is the probability density function of the received vector **y**_*u*_, conditioned on **s**_*u*_ and ${\hat{H}}_{u}$. Accordingly, the Euclidean distance between the received vector and all possible symbol transmissions is minimized by the ML detector. As noted, it is assumed that the transmitter and receiver have perfect CSI. At the transmitter, CSI is used to choose optimal *N*_t_ out of *N*_L_ LEDs to achieve the best minimum distance at the receiver side. Based on the Frobenius norm in (3.1), the ML detector evaluates the summation of the square of *N*_p_ observations and yields an optimal decision regarding the detected symbol transmitted. For simplicity in the analytical derivations of the symbol error ratio (SER) calculations,^3^ we consider the *N*_t_ × *N*_p_ MIMO system with *N*_p_ = 1.

As it is shown in [19], the simplified symbol error ratio (SER) for the *N*_t_ × 1 *M*-PAM optical SM system can be computed as

3.2 $$\text{SER}=\frac{2}{K}\sum_{r=1}^{K-1}Q\left(\frac{d_{r,r+1}^{u}}{2\sigma }\right),$$

where $d_{r,r+1}^{u}$ is the Euclidean distance between the *r*^th^ and (*r* + 1)^th^ adjacent constellation points for user *u*; *r* = 1, 2, …, *K* is the symbol index; *K* represents the combination of the spatial and constellation symbols with *K* = *MN*_*t*_; and $Q(x)=(1/\sqrt{2\pi })\int_{x}^{\infty }\exp^{-(t^{2}/2)}\;\text{d}t$.

It can be seen from (3.2), the error performance depends on the distance between the symbols and noise power. In order to solely investigate the effect of the similarities among multiple channels on the error performance of the *N*_t_ × 1 M-PAM system and propose a relationship between the channel gains and error performance, the additive noise contribution is neglected in the received signal of all users given by (2.1). When it is assumed that the noise is not present and $h_{u,{\text{LED}}_{u,1}}>h_{u,{\text{LED}}_{u,2}}>\ldots >h_{u,{\text{LED}}_{u,N_{\text{t}}}}$ where $h_{u,{\text{LED}}_{u,t}}\in {\hat{H}}_{u}$, the received signals, ${\tilde{y}}_{u}$, can be written as given in tables 1 and 2 for 2 × 1 and 4 × 1, 2-PAM optical SM systems, respectively. As the relation between the channel gains affects the ordering of the amplitude of the received signals, all the possible orders are considered in the given tables. For the sake of simplicity, the channel gain of the selected LEDs is normalized by $h_{u,{\text{LED}}_{u,1}}$ which is the maximum achieved channel gain for a given user location and the user index *u* is omitted. Accordingly, the channel gain relation calculations are performed for a single-user system with the normalized channel gain $h_{t}^{\prime }$.

**Table 1.** $\tilde{y}$ and relation between the normalized channel gains for 2 × 1, 2-PAM.

case	${\tilde{y}}_{1}$	${\tilde{y}}_{2}$	${\tilde{y}}_{3}$	${\tilde{y}}_{4}$	$h_{1}^{\prime }$	$h_{2}^{\prime }$	range
1	$Ah_{2}^{\prime }$	$Ah_{1}^{\prime }$	$3Ah_{2}^{\prime }$	$3Ah_{1}^{\prime }$	1	0.5	$h_{2}^{\prime }>(h_{1}^{\prime }/3)$
2	$Ah_{2}^{\prime }$	$3Ah_{2}^{\prime }$	$Ah_{1}^{\prime }$	$3Ah_{1}^{\prime }$	1	0.2	$h_{2}^{\prime }<(h_{1}^{\prime }/3)$

**Table 2.** $\tilde{y}$ and relation between the normalized channel gains for 4 × 1, 2-PAM.

case	${\tilde{y}}_{1}$	${\tilde{y}}_{2}$	${\tilde{y}}_{3}$	${\tilde{y}}_{4}$	${\tilde{y}}_{5}$	${\tilde{y}}_{6}$	${\tilde{y}}_{7}$	${\tilde{y}}_{8}$	$h_{1}^{\prime }$	$h_{2}^{\prime }$	$h_{3}^{\prime }$	$h_{4}^{\prime }$	range
1	$Ah_{4}^{\prime }$	$Ah_{3}^{\prime }$	$Ah_{2}^{\prime }$	$Ah_{1}^{\prime }$	$3Ah_{4}^{\prime }$	$3Ah_{3}^{\prime }$	$3Ah_{2}^{\prime }$	$3Ah_{1}^{\prime }$	1	0.83	0.67	0.5	$h_{4}^{\prime }>(h_{1}^{\prime }/3)$
2	$Ah_{4}^{\prime }$	$Ah_{3}^{\prime }$	$Ah_{2}^{\prime }$	$3Ah_{4}^{\prime }$	$Ah_{1}^{\prime }$	$3Ah_{3}^{\prime }$	$3Ah_{2}^{\prime }$	$3Ah_{1}^{\prime }$	1	0.6	0.4	0.27	$\left(h_{2}^{\prime }/3)<h_{4}^{\prime }<(h_{1}^{\prime }/3\right)$;
													$h_{3}^{\prime }>(h_{1}^{\prime }/3)$
3	$Ah_{4}^{\prime }$	$Ah_{3}^{\prime }$	$Ah_{2}^{\prime }$	$3Ah_{4}^{\prime }$	$3Ah_{3}^{\prime }$	$Ah_{1}^{\prime }$	$3Ah_{2}^{\prime }$	$3Ah_{1}^{\prime }$	1	0.54	0.28	0.23	$(h_{2}^{\prime }/3)<h_{4}^{\prime }$ ; $(h_{1}^{\prime }/3)<h_{2}^{\prime }$;
													$h_{3}^{\prime }<(h_{1}^{\prime }/3)$
4	$Ah_{4}^{\prime }$	$Ah_{3}^{\prime }$	$3Ah_{4}^{\prime }$	$Ah_{2}^{\prime }$	$Ah_{1}^{\prime }$	$3Ah_{3}^{\prime }$	$3Ah_{2}^{\prime }$	$3Ah_{1}^{\prime }$	1	0.73	0.42	0.19	$\left(h_{2}^{\prime }/3)>h_{4}^{\prime }>(h_{3}^{\prime }/3\right)$;
													$h_{3}^{\prime }>(h_{1}^{\prime }/3)$
5	$Ah_{4}^{\prime }$	$Ah_{3}^{\prime }$	$3Ah_{4}^{\prime }$	$Ah_{2}^{\prime }$	$3Ah_{3}^{\prime }$	$Ah_{1}^{\prime }$	$3Ah_{2}^{\prime }$	$3Ah_{1}^{\prime }$	1	0.67	0.28	0.16	$\left(h_{2}^{\prime }/3)>h_{4}^{\prime }>(h_{3}^{\prime }/3\right)$;
													$(h_{1}^{\prime }/3)<h_{2}^{\prime }$;
													$\left(h_{2}^{\prime }/3)<h_{3}^{\prime }<(h_{1}^{\prime }/3\right)$
6	$Ah_{4}^{\prime }$	$3Ah_{4}^{\prime }$	$Ah_{3}^{\prime }$	$Ah_{2}^{\prime }$	$Ah_{1}^{\prime }$	$3Ah_{3}^{\prime }$	$3Ah_{2}^{\prime }$	$3Ah_{1}^{\prime }$	1	0.78	0.56	0.11	$h_{4}^{\prime }<(h_{3}^{\prime }/3)$; $(h_{1}^{\prime }/3)<h_{3}^{\prime }$
7	$Ah_{4}^{\prime }$	$Ah_{3}^{\prime }$	$Ah_{2}^{\prime }$	$3Ah_{4}^{\prime }$	$3Ah_{3}^{\prime }$	$3Ah_{2}^{\prime }$	$Ah_{1}^{\prime }$	$3Ah_{1}^{\prime }$	1	0.19	0.15	0.11	$(h_{2}^{\prime }/3)<h_{4}^{\prime }$; $h_{2}^{\prime }<(h_{1}^{\prime }/3)$
$6^{*}$	$Ah_{4}^{\prime }$	$3Ah_{4}^{\prime }$	$Ah_{3}^{\prime }$	$Ah_{2}^{\prime }$	$Ah_{1}^{\prime }$	$3Ah_{3}^{\prime }$	$3Ah_{2}^{\prime }$	$3Ah_{1}^{\prime }$	1	0.6	0.43	0.09	$h_{4}^{\prime }<(h_{3}^{\prime }/3)$; $(h_{1}^{\prime }/3)<h_{3}^{\prime }$
8	$Ah_{4}^{\prime }$	$Ah_{3}^{\prime }$	$3Ah_{4}^{\prime }$	$3Ah_{3}^{\prime }$	$Ah_{2}^{\prime }$	$Ah_{1}^{\prime }$	$3Ah_{2}^{\prime }$	$3Ah_{1}^{\prime }$	1	0.67	0.11	0.07	$(h_{3}^{\prime }/3)<h_{4}^{\prime }$; $h_{3}^{\prime }<(h_{2}^{\prime }/3)$;
													$(h_{1}^{\prime }/3)<h_{2}^{\prime }$
9	$Ah_{4}^{\prime }$	$Ah_{3}^{\prime }$	$3Ah_{4}^{\prime }$	$Ah_{2}^{\prime }$	$3Ah_{3}^{\prime }$	$3Ah_{2}^{\prime }$	$Ah_{1}^{\prime }$	$3Ah_{1}^{\prime }$	1	0.25	0.17	0.06	$h_{4}^{\prime }<(h_{2}^{\prime }/3)$; $(h_{2}^{\prime }/3)<h_{3}^{\prime }$;
													$h_{2}^{\prime }<(h_{1}^{\prime }/3)$
10	$Ah_{4}^{\prime }$	$3Ah_{4}^{\prime }$	$Ah_{3}^{\prime }$	$Ah_{2}^{\prime }$	$3Ah_{3}^{\prime }$	$Ah_{1}^{\prime }$	$3Ah_{2}^{\prime }$	$3Ah_{1}^{\prime }$	1	0.67	0.28	0.06	$h_{4}^{\prime }<(h_{3}^{\prime }/3)$; $h_{3}^{\prime }<(h_{1}^{\prime }/3)$;
													$(h_{2}^{\prime }/3)<h_{3}^{\prime }$; $(h_{1}^{\prime }/3)<h_{2}^{\prime }$
11	$Ah_{4}^{\prime }$	$Ah_{3}^{\prime }$	$3Ah_{4}^{\prime }$	$3Ah_{3}^{\prime }$	$Ah_{2}^{\prime }$	$3Ah_{2}^{\prime }$	$Ah_{1}^{\prime }$	$3Ah_{1}^{\prime }$	1	0.2	0.05	0.03	$(h_{3}^{\prime }/3)<h_{4}^{\prime }$; $h_{3}^{\prime }<(h_{2}^{\prime }/3)$;
													$h_{2}^{\prime }<(h_{1}^{\prime }/3)$
12	$Ah_{4}^{\prime }$	$3Ah_{4}^{\prime }$	$Ah_{3}^{\prime }$	$3Ah_{3}^{\prime }$	$Ah_{2}^{\prime }$	$Ah_{1}^{\prime }$	$3Ah_{2}^{\prime }$	$3Ah_{1}^{\prime }$	1	0.67	0.13	0.027	$h_{4}^{\prime }<(h_{3}^{\prime }/3)$; $h_{3}^{\prime }<(h_{2}^{\prime }/3)$;
													$(h_{1}^{\prime }/3)<h_{2}^{\prime }$
$11^{*}$	$Ah_{4}^{\prime }$	$Ah_{3}^{\prime }$	$3Ah_{4}^{\prime }$	$3Ah_{3}^{\prime }$	$Ah_{2}^{\prime }$	$3Ah_{2}^{\prime }$	$Ah_{1}^{\prime }$	$3Ah_{1}^{\prime }$	1	0.2	0.05	0.025	$(h_{3}^{\prime }/3)<h_{4}^{\prime }$; $h_{3}^{\prime }<(h_{2}^{\prime }/3)$;
													$h_{2}^{\prime }<(h_{1}^{\prime }/3)$
$12^{*}$	$Ah_{4}^{\prime }$	$3Ah_{4}^{\prime }$	$Ah_{3}^{\prime }$	$3Ah_{3}^{\prime }$	$Ah_{2}^{\prime }$	$Ah_{1}^{\prime }$	$3Ah_{2}^{\prime }$	$3Ah_{1}^{\prime }$	1	0.67	0.11	0.022	$h_{4}^{\prime }<(h_{3}^{\prime }/3)$; $h_{3}^{\prime }<(h_{2}^{\prime }/3)$;
													$(h_{1}^{\prime }/3)<h_{2}^{\prime }$
13	$Ah_{4}^{\prime }$	$3Ah_{4}^{\prime }$	$Ah_{3}^{\prime }$	$3Ah_{3}^{\prime }$	$Ah_{2}^{\prime }$	$3Ah_{2}^{\prime }$	$Ah_{1}^{\prime }$	$3Ah_{1}^{\prime }$	1	0.2	0.033	0.007	$h_{4}^{\prime }<(h_{3}^{\prime }/3)$; $h_{3}^{\prime }<(h_{2}^{\prime }/3)$;
													$h_{2}^{\prime }<(h_{1}^{\prime }/3)$
$X^{*}$: case with two valid solutions.													

In the first case, it is assumed that $h_{2}^{\prime }>(h_{1}^{\prime }/3)$ whereas in the second case, the relation between $h_{1}^{\prime }$ and $h_{2}^{\prime }$ is $h_{2}^{\prime }<(h_{1}^{\prime }/3)$. The Euclidean distance between the adjacent symbols *r* and *r* + 1 can be obtained by $d_{r,r+1}={\tilde{y}}_{r+1}-{\tilde{y}}_{r}$, where ${\tilde{y}}_{r}$ and ${\tilde{y}}_{r+1}$ represent the *r*^th^ and (*r* + 1)^th^ received symbol in the absence of noise. In order to minimize the SER, the given Euclidean distances should be maximized. It is shown in [19] that the minimum SER is achieved when the two possible weakest links are equal. For example, $d_{1,2}=A(h_{1}^{\prime }-h_{2}^{\prime })$, $d_{2,3}=A(3h_{2}^{\prime }-h_{1}^{\prime })$ and $d_{3,4}=A(3h_{1}^{\prime }-3h_{2}^{\prime })$ for case 1 in table 1. Since, *d*_3,4_ > *d*_1,2_, *d*_2,3_, hence, the relation between the channel gains can be obtained as $A(h_{1}^{\prime }-h_{2}^{\prime })=A(3h_{2}^{\prime }-h_{1}^{\prime })$ and $h_{2}^{\prime }=0.5h_{1}^{\prime }$. Following the same steps, the relation of the channel gains can be obtained for all cases. Consequently, the received symbol constellation diagram given in tables 1 and 2 can only be constructed with the given relation between the normalized channel gains.

In order to obtain the SER performance, the following steps are taken: (i) $\tilde{y}$ values are obtained for each case based on the given channel gains in tables 1 and 2; (ii) for the sake of fair performance comparison, $\tilde{y}$ values are normalized by the average transmission power, which is obtained as $\left(10/4)({h_{1}^{\prime }}^{2}+{h_{2}^{\prime }}^{2}\right)$ and $\left(10/8)({h_{1}^{\prime }}^{2}+{h_{2}^{\prime }}^{2}+{h_{3}^{\prime }}^{2}+{h_{4}^{\prime }}^{2}\right)$ for 2 × 1 and 4 × 1, 2-PAM systems, respectively; (iii) the Euclidean distance between the adjacent symbols is obtained; and (iv) the simplified SER performance is found using (3.2). For the 2 × 1 system, a SER of 10^−3^ is achieved at a signal-to-noise ratio (SNR) of 16.4 dB for case 1 and at 17 dB for case 2. For the 4 × 1 system, the optimum SER is achieved for case 6 given in table 2. A SER of 10^−3^ is achieved at a SNR of 20.8 dB for case 6 and at 22.5 dB for case 1.

Based on the relation between the channel gains, the highest total received signal power out of *N*_t_ LEDs can be achieved for case 1 in both systems. For the 2 × 1, 2-PAM system, the minimum SER given in (3.2) is also achieved with case 1. However, this is not the case for the 4 × 1, 2-PAM system. Although case 1 does not achieve the optimum error performance for the 4 × 1, 2-PAM system, it can be used to find a general relation between the channel gains for an arbitrary number of transmission antenna, *N*_t_, and modulation order, *M*. To propose a general relation between the channel gains for an *N*_t_ × 1, *M*-PAM system, the following design criteria have been considered in this paper: (i) $h_{1}^{\prime }>h_{2}^{\prime }>\ldots >h_{N_{\text{t}}}^{\prime }$; and (ii) $I_{m^{\prime }}^{M}h_{1}^{\prime }<I_{m^{\prime }+1}^{M}h_{N_{\text{t}}}^{\prime }$ where *m*^′^ = 1, 2, …, *M* − 1.

According to the given criteria above and when noise is not present, the components of the received signal vector, $\tilde{y}$, are shown in figure 2, in ascending order.

When the same power level is multiplied with the channel gains $h_{1}^{\prime }>h_{2}^{\prime }>\ldots >h_{N_{\text{t}}}^{\prime }$, the received signal vector $\tilde{y}$ will be in ascending order. This can be seen from figure 2 for the vectors (i) from ${\tilde{y}}_{1}$ to ${\tilde{y}}_{N_{t}}$; (ii) from ${\tilde{y}}_{N_{\text{t}}+1}$ to ${\tilde{y}}_{2N_{\text{t}}}$; and so on. However, when there is an increment in the used power level, such as (i) from ${\tilde{y}}_{N_{\text{t}}}$ to ${\tilde{y}}_{N_{\text{t}}+1}$; and (ii) ${\tilde{y}}_{(M-1)N_{\text{t}}}$ to ${\tilde{y}}_{(M-1)N_{\text{t}}+1}$, there is a possibility that ${\tilde{y}}_{N_{\text{t}}+1}<{\tilde{y}}_{N_{\text{t}}}$ and ${\tilde{y}}_{(M-1)N_{\text{t}}+1}<{\tilde{y}}_{(M-1)N_{\text{t}}}$. In other words, there is a possibility that the signal with a higher power level can become smaller than the signal with a smaller power level as $3Ah_{N_{\text{t}}}^{\prime }<Ah_{1}^{\prime }$ and $(2M-1)Ah_{N_{\text{t}}}^{\prime }<(2M-3)Ah_{1}^{\prime }$. The received symbol constellation diagram given in ascending order can only be evaluated when the relation between the maximum and minimum channel gains of the selected *N*_t_ LEDs is

3.3 $$\frac{\left(2M-3\right)}{\left(2M-1\right)}h_{1}^{\prime }\leq h_{N_{\text{t}}}^{\prime }.$$

Based on the relation given for case 1 in tables 1 and 2, it can be concluded that the relation between $h_{1}^{\prime }$ and $h_{N_{\text{t}}}^{\prime }$ that satisfies (3.3) can be obtained by (3.4*a*). According to the considered design criteria, the relation of the normalized channel gains for the generalized *N*_t_ × 1, *M*-PAM system is written as given in (3.4*b*).

3.4*a* $$h_{N_{\text{t}}}^{\prime }=\frac{M-1}{M}h_{1}^{\prime }.$$

3.4*b* $$h_{t^{\prime }}^{\prime }=1-(t^{\prime }-1)\tilde{h}⟺\tilde{h}=\frac{\left(1-h_{N_{\text{t}}}^{\prime }\right)}{\left(N_{\text{t}}-1\right)},\;t^{\prime }=2,\ldots ,N_{\text{t}}.$$

## 4. Designing light-emitting diodes array structure

Existing light fixtures consist of a different number of LEDs depending on the manufacturer’s design. In this study, an LED array consisting of *N*_L_ LEDs is considered. The number of selected LEDs for SM transmission is assumed to be 2 (*N*_t_ = 2).

It is important to note that the main functionality of the LED luminaires is to provide illumination in the environment. Data transmission through LEDs is an additional functionality. Thus, the proposed transmitter structure should firstly provide the desired illumination level. Then, the performance of the data transmission should be taken into account.

**Figure 3.**
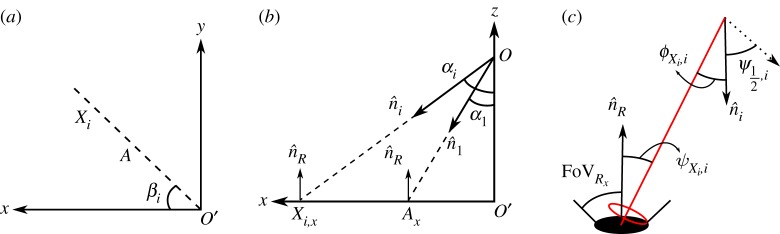
Illustration of orientation of LEDs. *β*_*i*_, *α*_*i*_ and ${\hat{n}}_{i}$ represent horizontal orientation, vertical orientation and normal of LED*i*, respectively; and ${\hat{n}}_{R}$ represents the receiver normal. The point *O* represents the location of the LED array on the ceiling. The points *A* and *X*_*i*_ represent the directed point of LED1 and LED*i* on the XY-plane. The points *A*_*x*_, *X*_*i*,*x*_ and *O*^′^ represent *x*-axis component of the points *A*, *X*_*i*_ and *O*, respectively. (*a*) XY-axes, (*b*) XZ-axes, (*c*) gain geometry. (Online version in colour.)

In order to provide the desired illumination level through the 4 m × 4 m × 3 m room, (i) the LEDs in the array should be oriented in different directions; and (ii) the channel gain of the LEDs at the directed point on the horizontal plane should be the same. A possible orientation of the LEDs that illuminate the environment and channel gain geometry are illustrated in figure 3. The optical channel gain at a point *X*_*i*_, which is the directed point of LED*i* on the horizontal plane, from LED*i* is expressed by the Lambertian reflection as [26]

4.1 $$h_{X_{i},i}=\frac{(m_{i}+1)A_{R_{x}}G_{R_{x}}}{2\pi {\left(|OX_{i}|\right)}^{2}}\cos^{m_{i}}(\phi_{X_{i},i})\cos(\psi_{X_{i},i})R,$$

where *m*_*i*_ = −ln2/ln(cos (Ψ_(1/2),*i*_)) is the Lambertian order and depends on the semi-angle of the LED*i*, Ψ_(1/2),*i*_; |*OX*_*i*_| represents the distance between points *O* and *X*_*i*_; $\phi_{X_{i},i}$ is the divergence angle from LED*i* to the receiver at the point *X*_*i*_ based on the normal of the LED*i*; $\psi_{X_{i},i}$ is the incidence angle from LED*i* to receiver at the point *X*_*i*_ based on the normal of the receiver; $G_{R_{x}}$ is the optical filter gain; $A_{R_{x}}$ is the area of the receiver; and $R=\text{rect}(\psi_{X_{i},i}\;/\;{\text{FOV}}_{R_{x}})$ is 0 or 1 according to the ratio of the incidence angle and FOV of the receiver, ${\text{FOV}}_{R_{x}}$. If the absolute value of the ratio is smaller than or equal to 1, the rect function gives 1 and otherwise, it gives 0. Following the rationale and justification as provided in [16], reflected optical paths can be neglected. Thus, only LoS links are considered in this paper.

When the room height (|*OO*^′^|), semi-angle ($\Psi_{\frac{1}{2},1}$) and horizontal orientation (*β*_1_) of LED1 are assumed to be known and ${\text{FOV}}_{R_{x}}=90^{\circ }$, the vertical orientation of LED1, as depicted in figure 3, can be computed as follows:

— *Consider $h_{O^{\prime },1}=(1/k)h_{A,1}$ to find *α*_1_ which is considered to be larger than 0° in order to break the symmetry in the room,*
  4.2 $$\frac{(m_{1}+1)A_{R_{x}}G_{R_{x}}}{2\pi {\left(|OO^{\prime }|\right)}^{2}}\cos^{m_{1}}(\phi_{O^{\prime },1})\cos(\psi_{O^{\prime },1})R\Leftrightarrow \frac{1}{k}\frac{(m_{1}+1)A_{R_{x}}G_{R_{x}}}{2\pi {\left(|OA|\right)}^{2}}\cos^{m_{1}}(\phi_{A,1})\cos(\psi_{A,1})R,$$
  where $\phi_{A,1}=\psi_{O^{\prime },1}=0^{\circ }$; $\phi_{O^{\prime },1}=\psi_{A,1}=\alpha_{1}$; and cos (*α*_1_) = |*OO*^′^|/|*OA*|. *R* = 1 in both equations as $\psi_{O^{\prime },1},\psi_{A,1}<{\text{FOV}}_{R_{x}}$. Thus, (4.2) can be written as
  4.3*a* $$\frac{\cos(\alpha_{1})}{k\frac{|OO^{\prime }{|}^{2}}{\cos^{2}(\alpha_{1})}}=\frac{\cos^{m_{1}}(\alpha_{1})}{|OO^{\prime }{|}^{2}}⟹(1/k)=\cos^{m_{1}-3}(\alpha_{1})⟹\cos(\alpha_{1})={\left(1/k\right)}^{\left(1/(m_{1}-3)\right)},$$
  and
  4.3*b* $$\alpha_{1}=\arccos{\left(\frac{1}{k}\right)}^{\left(1/\left(m_{1}-3\right)\right)}.$$

**Figure 4.**
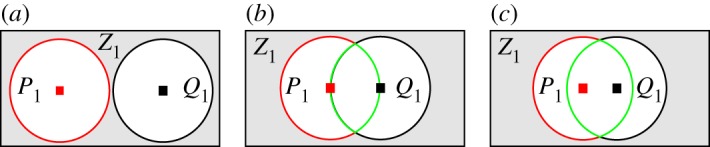
Cases for directed point of LEDs on the horizontal (receiver) plane. The centre of the sets *P*_1_ and *Q*_1_ represent the normalized channel gains ${\tilde{h}}_{j}=1$ and ${\tilde{h}}_{j-1}=1$, respectively. The set *P*_1_ represents ${\tilde{h}}_{j}>1/k$; *Q*_1_ represents ${\tilde{h}}_{j-1}>1/k$; and *Z*_1_ represents ${\tilde{h}}_{j},{\tilde{h}}_{j-1}<1/k$. (*a*) Case 1, (*b*) Case 2, (*c*) Case 3. (Online version in colour.)

According to (3.3), the channel gain of LED*N*_t_ should be larger than 1/*k* ∈ {1/3, 5/7, 13/15} of the channel gain of LED1 to support 2-PAM, 4-PAM or 8-PAM, respectively. In figure 4, three different cases are considered to decide how to chose the directed point of LEDs, in other words, *α*_*i*_ and *α*_*i*+1_, in a horizontal orientation *β*_*i*_. In all three cases, the sets *P*_1_, *Q*_1_ and *Z*_1_ represent the normalized channel gains ${\tilde{h}}_{j}>1/k$, ${\tilde{h}}_{j-1}>1/k$ and ${\tilde{h}}_{j},{\tilde{h}}_{j-1}<1/k$, respectively. The intersection of sets *P*_1_ and *Q*_1_ is $P_{1}\cap Q_{1}=\emptyset$ for case 1 and $P_{1}\cap Q_{1}>0$ for cases 2 and 3. Inherently, $P_{1}\cap Q_{1}$ in case 3 is larger than case 2. However, consider that case 3 may result in an excessive number of LEDs being deployed in a single horizontal orientation. In this paper, case 2 is used to decide the directed point of LEDs in a horizontal orientation. In order to deploy LEDs based on case 2, the location where the maximum channel gain is decreased by 1/*k* is chosen as the directed point of another LED, as depicted in figure 4. Accordingly, once the orientation of LED1 is found, the characteristics of the remaining LEDs can be determined based on the ratio 1/*k*. The Lambertian order and orientation of the remaining LEDs can be obtained as follows:

— *Consider $(1/k)h_{A,1}=h_{X_{2},1}$, where *X*_2_ is the directed point of LED2 as depicted in figure 3, to find *α*_2_, (*α*_2_ > *α*_1_)*
  4.4 $$\frac{1}{k}\frac{(m_{1}+1)A_{R_{x}}G_{R_{x}}}{2\pi {\left(|OA|\right)}^{2}}\cos^{m_{1}}(\phi_{A,1})\cos(\psi_{A,1})R\Leftrightarrow \frac{(m_{1}+1)A_{R_{x}}G_{R_{x}}}{2\pi {\left(|OX_{2}|\right)}^{2}}\cos^{m_{1}}(\phi_{X_{2},1})\cos(\psi_{X_{2},1})R,$$
  where *ϕ*_*A*,1_ = 0°; *ψ*_*A*,1_ = *α*_1_; $\phi_{X_{2},1}=\alpha_{2}-\alpha_{1}$; $\psi_{X_{2},1}=\alpha_{2}$; and cos (*α*_2_) = |*OO*^′^|/|*OX*_2_|. Moreover, $\psi_{A,1},\psi_{X_{2},1}<{\text{FOV}}_{R_{x}}$. Therefore, (4.4) can be written as
  4.5*a* $$\frac{1}{k}\cos^{3}(\alpha_{1})=\cos^{m_{1}}(\alpha_{2}-\alpha_{1})\cos^{3}(\alpha_{2})⟹{\left(\frac{1}{k}\frac{\cos^{3}(\alpha_{1})}{\cos^{3}(\alpha_{2})}\right)}^{\left(1/m_{1}\right)}=\cos(\alpha_{2}-\alpha_{1}),$$
  and
  4.5*b* $$\arccos\left({\left(\frac{1}{k}\frac{\cos^{3}(\alpha_{1})}{\cos^{3}(\alpha_{2})}\right)}^{\left(1/m_{1}\right)}\right)=(\alpha_{2}-\alpha_{1}),$$
  where the difference between the orientation angle of LEDs can be generalized by replacing subscripts 1 and 2 with *j* − 1 and *j*, respectively, in (4.5*b*) as
  4.6 $$\arccos\left({\left(\frac{1}{k}\frac{\cos^{3}(\alpha_{j-1})}{\cos^{3}(\alpha_{j})}\right)}^{\left(1/\left(m_{j-1}\right)\right)}\right)=(\alpha_{j}-\alpha_{j-1}).$$
— *To provide a uniform illumination level, consider $h_{A,1}=h_{X_{j},j}$ and find *m*_*j*_. It can be said that the divergence and incidence angles are $\phi_{A,1}=\phi_{X_{j},j}=0^{\circ }$ and $\psi_{X_{j},j}=\alpha_{j}$, respectively; and cos (*α*_*j*_) = |*OO*^′^|/|*OX*_*j*_|. The Lambertian order of LED*j* can be calculated by using (4.1) as follows:*
  4.7*a* $$\frac{\left(m_{1}+1)\cos(\alpha_{1}\right)}{|OA{|}^{2}}=\frac{\left(m_{j}+1)\cos(\alpha_{j}\right)}{|OX_{j}{|}^{2}}⟹\frac{\left(m_{1}+1)\cos^{3}(\alpha_{1}\right)}{|OO^{\prime }{|}^{2}}=\frac{\left(m_{j}+1)\cos^{3}(\alpha_{j}\right)}{|OO^{\prime }{|}^{2}},$$
  and
  4.7*b* $$m_{j}=\frac{\left(m_{1}+1)\cos^{3}(\alpha_{1}\right)}{\cos^{3}(\alpha_{j})}-1.$$
  In this work, the semi-angle of the LEDs is chosen from a finite set of [1°, 60°] with a 1° resolution. Based on (4.5*b*), *α*_2_ is a function of *α*_1_ where *α*_1_ is a function of *m*_1_. An integer-valued semi-angle for LED{*j* > 1} is found by
  4.8*a* $$\begin{array}{rl} \underset{\alpha_{j},m_{j}}{\text{minimize}} & \;{\left(\Psi_{(1/2),j}-\text{round}(\Psi_{(1/2),j})\right)}^{2} \end{array}$$
  4.8*b* $$\begin{array}{rl} \text{subject to} & \;(4.3b),(4.6),(4.7b),\;\forall j, \end{array}$$
  4.8*c* $$\begin{array}{rl} & \;\theta^{\circ }>\alpha_{j}>\alpha_{j-1}>0^{\circ },\;\forall j>1, \end{array}$$
  4.8*d* $$\begin{array}{rl} & \;\alpha_{j}-\alpha_{j-1}>\Psi_{\frac{1}{2},j-1},\;\forall j>1, \end{array}$$
  where the function round (.) rounds a value to its lower or upper integer number. The constraint given as *θ* in (4.8*c*) ensures that the orientation of the LED*j* is not directed to one of the side walls of the environment, and it is obtained by $\arctan\;(({\text{x}}_{\text{dim}}/2)/{\text{z}}_{\text{dim}})$, where x_dim_ and z_dim_ represent *x*-dimension and *z*-dimension (height) of the room. The reason behind dividing the *x*-dimension to 2 is due to the assumption that the LED array will be located in the middle of the room. Similarly, the constraint given in (4.8*d*) ensures that the directed point on the horizontal plane of the next LED is in the coverage area of the previous LED in order to have overlapping cells, as shown in figure 4*b*. The orientation and Lambertian order, inherently the semi-angle, of LED {j>1} at one horizontal resolution can be obtained by (4.8). Once the characteristics of the LEDs at *β* = 0° are found, the characteristics of the remaining LEDs can be obtained by shifting the horizontal orientation by *β*_*b*_ which represents the considered horizontal resolution. The LED characteristics for the horizontal orientation *β* = 0° are given in table 3 when the modulation order for transmit structure design^4^ *M*_*D*_ is considered as *M*_*D*_ = 2 and as *M*_*D*_ = 8.

**Table 3.** LED characteristics for *M*_*D*_ = 2 and *M*_*D*_ = 8 when *β* = 0°.

	*M*_*D*_ = 2			*M*_*D*_ = 8							
LED*j*	1	2	3	1	2	3	4	5	6	7	8
*α*_*j*_	17.3°	31°	42.9°	11.5°	17.7°	22.8°	27.2°	31°	34.4°	37.5°	40.3°
Ψ_(1/2),*j*_	13°	11°	9°	21°	20°	19°	18°	17°	16°	15°	14°

## 5. Light-emitting diodes selection for transmission

The relation between the channel gains given in (3.4) satisfies the minimum SER for an optical SM system with the considered design criteria when there is a single user. In single user systems, only one LED is activated per transmission period. However, when there is more than one user present in the system as depicted in figure 1, the number of activated LEDs will be equal to the number of users during a transmission period. Therefore, the error performance of the SM system is limited by not only the channel similarities of LEDs selected for a user but also among the set of LEDs that are selected for all users. Inherently, the SER performance of the multi-user system is limited by interference, instead of noise.

As noted, the set of LEDs selected for a user u is $L^{u}≜\{{\text{LED}}_{u,t}\}$. In order to prevent the selection of the same LED for more than one user, it can be said that $L^{1}\cap L^{2}\cap \ldots \cap L^{N_{\text{u}}}=\emptyset$ and $N_{\text{L}}⊃\{L^{1}\cup L^{2}\cup \ldots \cup L^{N_{\text{u}}}\}$.

For a single user system, *N*_u_ = 1, the transmit LED selection process used in this study is based on (3.4*b*). Firstly, the LED that provides the highest channel gain for a given user location is chosen as ${\text{LED}}_{1,1}\leftarrow \arg\max_{i}(h_{1,i})$. Then, based on the considered number of transmit LEDs, *N*_t_, and constellation size, *M*, the set $L^{1}$ is constructed in a way that satisfies $h_{1,{\text{LED}}_{1,1}}>h_{1,{\text{LED}}_{1,2}}>\ldots >h_{1,{\text{LED}}_{1,N_{\text{t}}}}$ and (3.3) by using (3.4). As having the exact same values for the normalized channel gains obtained by (3.4*b*) may not possible for all receiver locations, the LEDs that have the closest normalized gain to the values obtained by (3.4*b*) is chosen as follows:

$${\text{LED}}_{1,t}\leftarrow \arg\min_{i}{\left(\frac{h_{1,i}}{h_{1,{\text{LED}}_{1,1}}}-h_{t}^{\prime }\right)}^{2}\;t>1,\forall i,$$

where if there are several LEDs that have the same value, then, one of the LEDs is chosen randomly.

For a multi-user system, a signal-to-interference ratio (SIR) threshold *γ* in decibels is considered to decide the sets of LEDs that are going to be used for a user *u* and a user $\hat{u}\neq u$. Accordingly, the ratio of the channel gain from the LEDs in the set $L^{\hat{u}}$ for a user $\hat{u}$ to the user *u* should exceed *γ*. In other words:

5.1 $$\gamma \leq 10\log_{10}\left(\frac{h_{u,L^{u}}}{h_{u,L^{\hat{u}}}}\right)\;\forall u,\hat{u},$$

where $h_{u,L^{\hat{u}}}$ represents the channel gain of the LEDs in the set $L^{\hat{u}}$ at the location of the user *u*, and obtained by (4.1).

In general, a multi-user system needs a time and/or frequency-domain scheduler, and the system performance depends on the considered scheduling metrics such as proportional fair, max-min, min-max, etc. In this study, the objective is to evaluate the multi-user performance of the proposed LED array structure in a single transmission time interval. Therefore, instead of the performance of a system with a fixed number of users, the multi-user capability of the proposed LED array structure is examined. Accordingly, a randomly located user is considered as the first user, *u* = 1, and the steps given for a single user system are followed to obtain the *N*_t_-element set $L^{1}$. The LEDs that are not element of $L^{1}$ and satisfy the given SIR threshold in (5.1) are used to construct a set ${\hat{L}}^{\gamma }$. Then, an LED in the set ${\hat{L}}^{\gamma }$ is randomly chosen as the LED_2,1_. If the set $L^{2}$ can be constructed in a way that satisfies $h_{2,{\text{LED}}_{2,1}}>h_{2,{\text{LED}}_{2,2}}>\ldots >h_{2,{\text{LED}}_{2,N_{\text{t}}}}$ and (3.3) by using (3.4), then, *u* is considered as *u* = 2 and ${\hat{L}}^{\gamma }$ is updated by neglecting (i) the LEDs in $L^{u}$; and (ii) LEDs that do not satisfy the SIR threshold given as in (5.1). The same steps are followed until either ${\hat{L}}^{\gamma }=\emptyset$ or the number of the random LED selection from the set ${\hat{L}}^{\gamma }$ has reached *N*_L_/*N*_t_. Thereafter, the number of users that the given LED array structure can transmit to during a single transmission time interval is obtained for a given random location of the first user.

## 6. System simulation results

In the computer simulations, the system model given in [16] is used with some modifications. This is a 4 × 4 MIMO-optical SM system where *N*_t_ = 4 LEDs are located 0.6 m apart from each other and *N*_p_ = 4 PDs are located on the corners of a square with a side length of 0.1 m, as explained in detail in [16]. ${\text{FOV}}_{{\text{R}}_{\text{x}}}$ is considered as 15° for all PDs in [16]. However, in order to investigate the BER performance for the entire room, a larger ${\text{FOV}}_{{\text{R}}_{\text{x}}}$ is needed. Therefore, the system model given in [16] is used with a ${\text{FOV}}_{{\text{R}}_{\text{x}}}$ modification in this study. Accordingly, all the PDs are pointed upwards and assumed to have a 45° FOV (${\text{FOV}}_{R_{x}}=45^{\circ }$). The receiver height is considered as 0.75 m. Without loss of generality, the optical filter gain $G_{R_{x}}$ and the area of the receiver $A_{R_{x}}$ are taken as 1 and 1 cm^2^, respectively, to simplify the analysis. The transmitter is assumed to have a structure as described in table 3 for *M*_*D*_ = 2 and *M*_*D*_ = 8. Different horizontal resolutions *β*_*b*_ are considered. According to the considered *β*_*b*_, the LED array consists of *N*_L_ = *J*(360/*β*_*b*_) LEDs where *J* is equal to 3 for *M*_*D*_ = 2 and 8 for *M*_*D*_ = 8. The room is divided into 40×40 pixels where the receiver is located at the centre of each pixel. This is equivalent to sliding the square receiver through the room. To obtain the average BER performance, randomly generated spatial and constellation symbol sets are iterated 10^5^ times for each receiver location. The SNR is defined based on the received signal power. Additionally, as noted earlier, only the LoS link is considered in the channel gain calculation.

**Figure 5.**
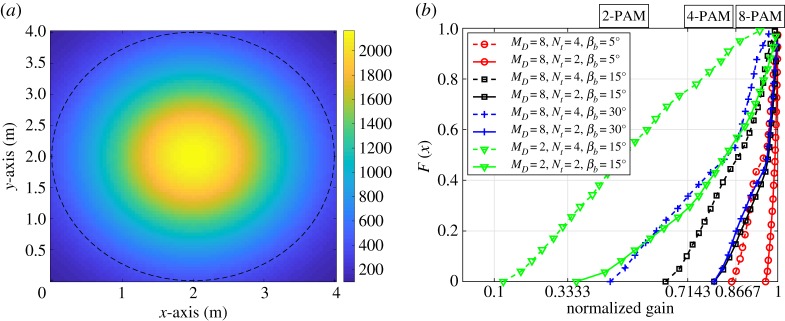
(*a*) The received horizontal illuminance level in lux [lx] on the receiver plane in the room when *M*_*D*_ = 8. The dashed line shows the border of the required level of 400 lx. (*b*) Normalized channel gain distribution of LED_1,2_ and LED_1,4_ in case of *N*_t_ = 2 and *N*_t_ = 4 when *M*_*D*_ = 2 and *M*_*D*_ = 8. (Online version in colour.)

As noted, the main functionality of the LED luminaries is to provide a required illumination level in the environment. For an indoor environment, 400 lx is considered as the required illumination level for reading purposes [27]. The spatial illuminance distribution on the receive plane is shown in figure 5 when *M*_*D*_ = 8 is chosen as the LED array design parameter. The required illumination level of 400 lx is provided at above 77% of the receive plane. This indicates that the proposed LED array structure satisfies the required illumination level in most of the room.

In figure 5*b*, the cumulative distribution function (CDF) of the normalized channel gain of the ${\text{LED}}_{1,N_{t}}$ is shown for *N*_t_ = 2 and *N*_t_ = 4 when the LED array is designed for *M*_*D*_ = 2 and *M*_*D*_ = 8. The CDF is defined as the function *F*(*x*) refers to the probability of the random variable *X* taking on values less than or equal to *x*. The normalized channel gain is obtained by dividing the achieved channel gain of the LEDs with the gain of LED_1,1_, which is the maximum achieved gain for a given receiver location. Hence, based on (3.3), the channel gain of ${\text{LED}}_{1,N_{\text{t}}}$ should be larger than 1/3, 5/7 and 13/15 of the channel gain of the LED_1,1_ for 2-PAM, 4-PAM and 8-PAM, respectively. Also, the channel gain of ${\text{LED}}_{1,N_{\text{t}}}$ should be smaller than 1 to satisfy $h_{1}^{\prime }>h_{N_{\text{t}}}^{\prime }$.

According to figure 5*b*, when the LED array is designed for *M*_*D*_ = 2, the inequality given by (3.3) is satisfied for around 99%, 69% and 40% of the illuminated area when the system with *N*_t_ = 2 and *β*_*b*_ = 15° is considered with 2-PAM, 4-PAM and 8-PAM optical SM transmission, respectively. However, when the system with *N*_t_ = 4 is considered, 2-PAM transmission can be supported by 75% of the illuminated area for the given transmitter structure. When the design is carried out for *M*_*D*_ = 8 with *β*_*b*_ = 15°, it is shown that 8-PAM optical SM transmission can be used in around 85% and 50% of the illuminated area for *N*_t_ = 2 and *N*_t_ = 4, respectively. In order to provide 8-PAM transmission for all of the illuminated area, a smaller *β*_*b*_ should be considered. For example, when the structure is designed based on *M*_*D*_ = 8 with *β*_*b*_ = 5°, 8-PAM optical SM transmission can be supported by 99% for both *N*_t_ = 2 and *N*_t_ = 4.

**Figure 6.**
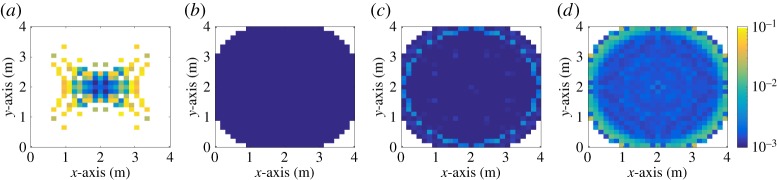
Average BER performance inside the room when the transmitter structure is designed based on *M*_*D*_ = 8, *β*_*b*_ = 15° and the received SNR is considered as 30 dB. White areas represent either BER higher than 0.1 or no channel gain. (*a*) *N*_t_ = 4, 2-PAM [16], (*b*) *N*_t_ = 2, 2-PAM, (*c*) *N*_t_ = 2, 4-PAM, (*d*) *N*_t_ = 2, 8-PAM. (Online version in colour.)

In figure 6, the average BER performance of different optical SM systems is shown when the transmitter structure is designed based on *M*_*D*_ = 8, *β*_*b*_ = 15° and the received SNR is considered as 30 dB. The reasons for considering a high and fixed received SNR value in figure 6 are to show (i) how channel similarity affects the error performance; and (ii) how the proposed transmitter structure mitigates the similarity effects all around the room. In figure 6*a*, the performance of the 4 × 4 MIMO-optical SM system proposed in [16] is shown when 2-PAM is used as the modulation order and ${\text{FOV}}_{R_{x}}=45^{\circ }$. The spectral efficiency of the system in figure 6*a* is 3 bits/s/Hz. As it can be seen from figure 6*a*, the system achieves a low BER performance only at the centre of the room.

When the proposed transmit structure is considered in a 2 × 4 MIMO-optical SM system with 4-PAM, the same spectral efficiency is achieved notably in a larger area than the system given in [16] as shown in figure 6*c*. In figure 6*b*, it can be seen that the spectral efficiency of 2 bits/s/Hz can be achieved error-free in the most of the room. Also, it is shown in figure 6*d* that the BER performance of the system with 8-PAM is slightly above 10^−3^. A drop of BER performance in figure 6*c*,*d* around the edge of the illuminated area is due to the considered horizontal resolution *β*_*b*_. It can be mitigated by decreasing *β*_*b*_ in order to increase the intersection of channel gain sets around the edge of the illuminated area.

**Figure 7.**
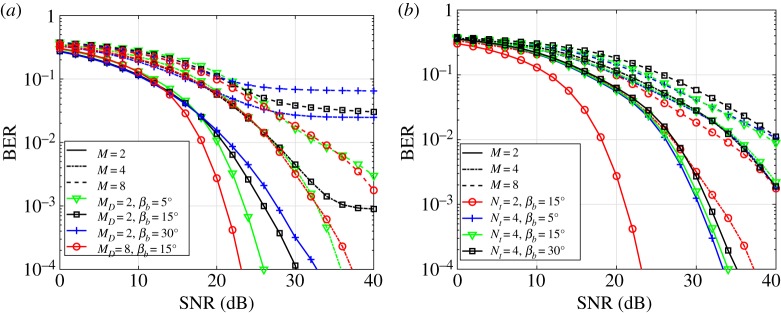
The BER performance of the system with different *M*, *β*_*b*_, *M*_*D*_ and *N*_*t*_ values. The solid, dash-dotted and dashed lines represent *M* = 2, *M* = 4 and *M* = 8, respectively. (*a*) *N*_t_ = 2. The different colours/markers represent a combination of *M*_*D*_ and *β*_*b*_. For example, the blue/+ dashed graph represents the performance of the system with *M* = 8, *M*_*D*_ = 2 and *β*_*b*_ = 15°. (*b*) *M*_*D*_ = 8. The different colours/markers represent a combination of *N*_t_ and *β*_*b*_. For example, the black/□ dash-dotted graph represents the performance of the system with *M* = 4, *N*_t_ = 4 and *β*_*b*_ = 30°. (Online version in colour.)

In order to understand how the design parameters affect the overall BER performance, SNR versus BER graphs are shown for different parameter values of *M* and *N*_t_. In figure 7*a*, the performance of different *M*, *β*_*b*_ and *M*_*D*_ values are considered when *N*_t_ = 2 LEDs are used for optical SM transmission. According to figure 7*a*, increasing the horizontal resolution angle *β*_*b*_ generates an error floor when higher modulation orders are used. As it can be seen from the figure, when *M*_*D*_ = 2, all the considered *β*_*b*_ values can achieve a BER of 10^−3^ below an SNR of 30 dB for *M* = 2. However, the designed array structure for *M*_*D*_ = 2 may not achieve a sufficient BER performance to apply FEC channel coding for higher modulation orders such as *M* = 4 and *M* = 8. When the plots for *M*_*D*_ = 2, *M* = 4, *β*_*b*_ = 30° (blue/+, dashed-dotted line) and *M*_*D*_ = 2, *M* = 4, *β*_*b*_ = 15° (black/□, dashed-dotted line) in figure 7*a* are compared, it can be seen that 4-PAM can be supported even if the array structure is designed for *M*_*D*_ = 2-PAM. A BER of 10^−3^ is achieved for 4-PAM when *β*_*b*_ = 15° and the received SNR is 35 dB. However, this is not the case for *β*_*b*_ = 30° as it has an error floor around 5 × 10^−2^. This is because the possibility of satisfying the relation of the channel gain and minimum error probability is limited, as depicted in figure 5*b*.

In figure 7*b*, the performance of different *M*, *β*_*b*_ and *N*_t_ values are considered when the LED array structure is designed for *M*_*D*_ = 8. Based on the given figure, SNR values above 30 dB and 40 dB are needed to provide more than 4 bits/s/Hz and 5 bits/s/Hz, respectively, at the BER of 10^−3^.

**Figure 8.**
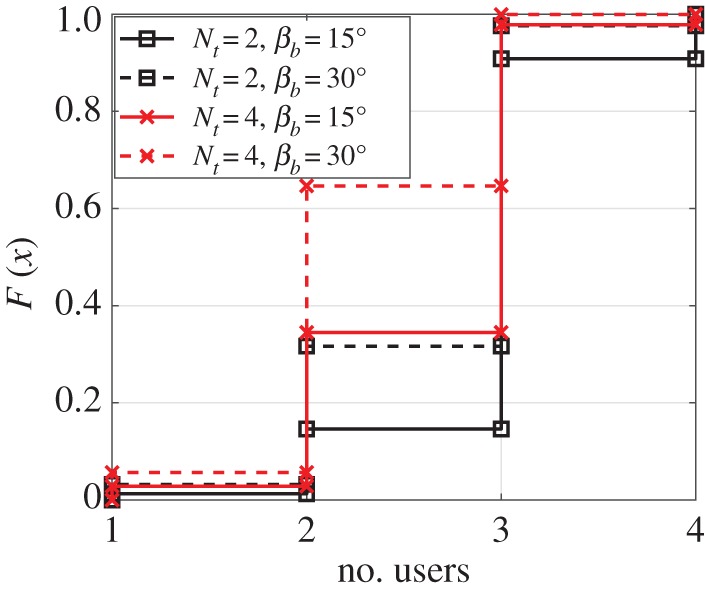
The CDF of the number of users that can be used for transmission when *M*_*D*_ = 8 is used to design the LED array. The SIR threshold *γ* is chosen as 32 dB and *M* = 2 is considered for PAM. (Online version in colour.)

In order to understand how the given LED array structure supports multi-user transmission, the average number of users that satisfies (3.4*b*) and the given SIR threshold *γ* is shown in figure 8 for different *N*_t_, *M* and *β*_*b*_ values. The LED array is designed based on *M*_*D*_ = 8. Therefore, the LED array consists of *N*_L_ = 192 LEDs when *β*_*b*_ = 15° and *N*_L_ = 96 LEDs when *β*_*b*_ = 30°. Based on the single user error performance, a 32 dB SIR is chosen as the threshold *γ*. As the performance of the multi-user system becomes interference-limited, it is assumed that the interference power is much greater than the noise power.

The set of possible LEDs for a user *u* is obtained by neglecting (i) the LEDs that are already chosen for transmission of another user; (ii) the LEDs that cause the SIR level to drop below the set threshold *γ*; and (iii) the LEDs do not satisfy (3.4*b*). To obtain an average value for the possible number of users that the given system can support, users are randomly located and this process is iterated 10^5^ times.

According to figure 8, the system with *M* = 2 and *N*_t_ = 2 can support an average of 2.9 and 2.68 users during a single transmission period for *β*_*b*_ = 15° and *β*_*b*_ = 30°, respectively. When *N*_t_ = 4 is used, the average number of users is 2.64 for *β*_*b*_ = 15° and 2.3 for *β*_*b*_ = 30°. Hence, the average achievable system throughput is 5.85 bits/s/Hz when *N*_t_ = 2 and *β*_*b*_ = 15°; 5.34 bits/s/Hz when *N*_t_ = 2 and *β*_*b*_ = 30°; 7.93 bits/s/Hz when *N*_t_ = 4 and *β*_*b*_ = 15°; and 6.9 bits/s/Hz when *N*_t_ = 4 and *β*_*b*_ = 30°. It is important to note that the given average number of users can be supported without the need for a power allocation algorithm or a transmit precoding technique to separate the multiple channels. Inherently, employing adaptive power allocation and precoding can improve multi-user capability of the proposed transmitter structure.

## 7. Conclusion

In this paper, a novel LED array structure is proposed to enable reliable optical MIMO-SM transmission without needing a precoder, power allocation algorithm or additional optics at the receiver. A single LED array, which consists of multiple LEDs with different characteristics, is used for both illumination and simultaneous data transmission. The relationship between error probability and channel similarity is provided for an *M*-ary PAM-SM system. Based on the given relation, the LED array is designed to separate multiple channels. It has been demonstrated that channel similarities can be reduced by manipulating the transmitter geometry and SM can be employed for IM/DD systems. Simulation results show that the proposed structure can achieve sufficient BER performance to apply forward error correction (FEC) channel coding for a single user inside a 4 m × 4 m × 3 m room. It is also shown that the proposed structure can reliably serve spatially separated users. We believe that the given analysis in this work can be efficiently used for designing an LED array, which can be used for both illumination and data transmission. Further enhancements on the number of users that can be served simultaneously may be achieved by employing angle diversity receivers with non-imaging PDs. Also, a similar BER and data rate performance could be achieved with a simple transmitter and a complex receiver structure. A combined structure design for the transmitter and receiver with simple components as well as performance comparisons with systems that employ a simple transmitter along with imaging MIMO and/or complex receiver structures will be considered as the future study items.

## Supplementary Material

TeX/LaTeX Class file

## Supplementary Material

Acronyms file

## Footnotes

1 As correlation is a statistical measure that quantifies the interdependency of random variables. This terminology is applicable to RF systems where there is a lot of randomness in the channel. However, in IM/DD systems, the term similarity is used to represent the relationship between the optical channel gains that have strong deterministic components.

2 Interested readers are referred to [4] for a comprehensive overview and detailed description of SM-MIMO systems.

3 The error performance and transmitter design works are carried out with a single PD scenario, in order to be analytically tractable. However, in the simulation part, a MIMO SM system is considered to have a fair performance comparison with the benchmark system given in [17].

4 *M*_*D*_ is used as the structure design parameter. For example, the LED array can be designed to support 8-PAM, and 2-PAM can be used in the performance evaluations. In this case, *M*_*D*_ = 8 and *M* = 2. Therefore, *M* in (3.3) and (3.4*a*) is replaced with *M*_*D*_ in the transmit structure design process.

## Data accessibility

Data sharing not applicable to this article as no datasets were generated.

## References

[1] Foschini G, Gans M 1998 On limits of wireless communications in a fading environment when using multiple antennas. Wireless Pers. Commun. 6, 311–335. (10.1023/A:1008889222784)

[2] Zheng L, Tse DNC 2003 Diversity and multiplexing: a fundamental tradeoff in multiple-antenna channels. IEEE Trans. Inf. Theory 49, 1073–1096. (10.1109/TIT.2003.810646)

[3] Joham M, Utschick W, Nossek J 2005 Linear transmit processing in MIMO communications systems. IEEE Trans. Signal Process. 53, 2700–2712. (10.1109/TSP.2005.850331)

[4] Di Renzo M, Haas H, Ghrayeb A, Sugiura S, Hanzo L 2014 Spatial modulation for generalized MIMO: challenges, opportunities, and implementation. Proc. IEEE 102, 56–103. (10.1109/JPROC.2013.2287851)

[5] Mesleh R, Haas H, Ahn CW, Yun S 2006 Spatial modulation—a new low complexity spectral efficiency enhancing technique. In *1st Int. Conf. Commun. and Netw. in China*, pp. 1–5.

[6] Renzo MD, Haas H 2012 Bit error probability of SM-MIMO over generalized fading channels. IEEE Trans. Veh. Technol. 61, 1124–1144. (10.1109/TVT.2012.2186158)

[7] Yang P, Guan YL, Xiao Y, Renzo MD, Li S, Hanzo L 2016 Transmit precoded spatial modulation: maximizing the minimum euclidean distance versus minimizing the bit error ratio. IEEE Trans. Wireless Commun. 15, 2054–2068. (10.1109/TWC.2015.2497692)

[8] Zeng L, O’Brien DC, Minh HL, Faulkner GE, Lee K, Jung D, Oh Y, Won ET 2009 High data rate multiple input multiple output (MIMO) optical wireless communications using white LED lighting. IEEE J. Sel. Areas Commun. 27, 1654–1662. (10.1109/JSAC.2009.091215)

[9] He C, Wang TQ, Armstrong J 2015 Performance of optical receivers using photodetectors with different fields of view in a MIMO ACO-OFDM system. J. Lightwave Technol. 33, 4957–4967. (10.1109/JLT.2015.2484385)

[10] Park KH, Ko YC, Alouini M 2013 On the power and offset allocation for rate adaptation of spatial multiplexing in optical wireless MIMO channels. IEEE Trans. Commun. 61, 1535–1543. (10.1109/TCOMM.2013.012913.110290)

[11] Ma H, Lampe L, Hranilovic S 2013 Robust MMSE linear precoding for visible light communication broadcasting systems. *Proc. IEEE Global Commun. Conf. Workshop*, pp. 1081–1086. Piscataway, NJ: IEEE.

[12] Cogalan T, Haas H, Panayirci E 2015 Power control-based multi-user Li-Fi using a compound eye transmitter. In *Proc. IEEE Global Commun. Conf.*, pp. 1–6. Piscataway, NJ: IEEE. 10.1098/rsta.2019.0195 PMC7062001 32114922

[13] Lian J, Brandt-Pearce M 2015 Distributed power allocation for multiuser MISO indoor visible light communications. In *Proc. IEEE Global Commun. Conf.*, pp. 1–7.

[14] Nuwanpriya A, Ho SW, Chen CS 2015 Indoor MIMO visible light communications: novel angle diversity receivers for mobile users. IEEE J. Sel. Areas Commun. 33, 1780–1792. (10.1109/JSAC.2015.2432514)

[15] Yu Z, Baxley R, Zhou G 2013 Multi-user MISO broadcasting for indoor visible light communication. *Proc. IEEE Int. Conf. Acoust., Speech, and Signal Process.* pp. 4849–4853.

[16] Fath T, Haas H 2013 Performance comparison of MIMO techniques for optical wireless communications in indoor environments. IEEE Trans. Commun. 61, 733–742. (10.1109/TCOMM.2012.120512.110578)

[17] Mesleh R, Elgala H, Haas H 2011 Optical spatial modulation. IEEE/OSA J. Opt. Commun. Netw. 3, 234–244. (10.1364/JOCN.3.000234)

[18] Popoola WO 2013 Merits and limitations of spatial modulation for optical wireless communications. In *Proc. 2nd Int. Workshop on Opt. Wireless Commun.*, pp. 152–156.

[19] Yesilkaya A, Cogalan T, Panayirci E, Haas H, Poor HV 2018 Achieving minimum error in MISO optical spatial modulation. In *Proc. IEEE Int. Conf. Commun.*, pp. 1–6.

[20] Lian J, Brandt-Pearce M 2017 Multiuser MIMO indoor visible light communication system using spatial multiplexing. J. Lightwave Technol. 35, 5024–5033. (10.1109/JLT.2017.2765462)

[21] Dambul KD, O’Brien D, Faulkner G 2011 Indoor optical wireless MIMO system with an imaging receiver. IEEE Photon. Technol. Lett. 23, 97–99. (10.1109/LPT.2010.2091627)

[22] Rajbhandari S *et al.* 2014 Imaging-MIMO visible light communication system using *μ*LEDs and integrated receiver. In *IEEE Global Commun. Conf. Workshop*, pp. 536–540.

[23] Wang TQ, Sekercioglu YA, Armstrong J 2012 Hemispherical lens based imaging receiver for MIMO optical wireless communications. In *IEEE Global Commun. Conf. Workshops*, pp. 1239–1243. Piscataway, NJ: IEEE.

[24] Danakis C, Afgani M, Povey G, Underwood I, Haas H 2012 Using a CMOS camera sensor for visible light communication. In *Proc. IEEE Global Commun. Conf. Workshops*, pp. 1244–1248.

[25] Han B, Hranilovic S 2018 A fixed-scale pixelated MIMO visible light communication system. IEEE J. Sel. Areas Commun. 36, 203–211. (10.1109/JSAC.2017.2774706)

[26] Kahn JM, Barry JR 1997 Wireless infrared communications. Proc. IEEE 85, 265–298. (10.1109/5.554222)

[27] European Standard Std. 2009 Lighting for indoor work places - EN 12464-1.

[28] Cogalan T, Haas H, Panayirci E 2019 A novel transmit array structure for optical spatial modulation. In *Proc. IEEE Int. Conf. Commun., Shanghai, China*, pp. 1–6. Piscataway, NJ: IEEE. 10.1098/rsta.2019.0195 PMC7062001 32114922

